# Supplementary material for: Cell-to-cell variability in inducible Caspase9-mediated cell death
Source: Cell Death Dis. 2022 Jan 10;13(1):34. doi: 10.1038/s41419-021-04468-z (PMC8748834; doi:10.1038/s41419-021-04468-z)
Supplement: Supplementary file 1 — Supplementary Information [file 41419_2021_4468_MOESM1_ESM.docx]

Supplementary Information for

**Cell-to-cell variability in inducible Caspase9-mediated cell death**

Yuan Yuan^1,2^, Huixia Ren^1^, Yanjun Li^1^, Shanshan Qin^1,2^, Xiaojing Yang^1,^*, Chao Tang^1,3,^*

**Affiliations**

^1^ Center for Quantitative Biology and Peking-Tsinghua Center for Life Sciences, Academy for Advanced Interdisciplinary Studies, Peking University, Beijing 100871, China.

^2^ School of Engineering and Applied Sciences, Harvard University, Cambridge, MA 02138, USA.

^3^ School of Physics, Peking University, Beijing 100871, China.

* Correspondence to: [tangc@pku.edu.cn](mailto:tangc@pku.edu.cn) and [xiaojing_yang@pku.edu.cn](mailto:xiaojing_yang@pku.edu.cn)

**This PDF file includes:**

Supplementary Text

Supplementary Figs. 1 to 6

**Other Supplementary Materials for this manuscript include the following:**

Supplementary Videos 1 to 5

**SUPPLEMENTARY INFORMATION**

In this paper, we constructed an iCaps9 system in which we can track the dynamics of iCasp9 dimerization and Caspase3 activation, and the cell fate simultaneously in single cells using live-cell imaging. Large heterogeneity in the clonal population of iCasp9 cells was observed. The heterogeneity not only manifested in the final cell fate of life and death, but also in the different surviving ways within surviving cells and the varied death timing within the dead cells.

**Further characterization of iCasp9 cell system.**

- For iCasp9 dimerization:

A significant fluorescence drop was observed after adding the inducer (Supplementary Video 1), while no fluorescent intensity change was observed either in microtubule-mCherry cells with inducer addition (Supplementary Fig. 1b) or in iCasp9-mCherry cells without inducer addition (Supplementary Fig. 1c).

- For FRET reporter:

Most unperturbed iCasp9 cells were alive with a low FRET Ratio (i.e. the FRET reporter within cells was intact without Caspase3 activation, Supplementary Fig. 1e and Supplementary Video 2), while most iCasp9 cells treated with 0.25 nM inducer died with high FRET Ratios (i.e. the FRET reporter was cleaved by activated Caspase3, Supplementary Fig. 1f and Supplementary Video 3). These results indicated that the FRET Ratio indeed is a good reporter for cell fate. To have the potential to scale up, we further validated that the FRET reporter can also report cell fate accurately through flow cytometry measurements (Supplementary Fig. 2a).

**Supplementary Fig. 1 Validation of iCasp9 cell system. a,** The iCasp9 activation dynamics (red curve in right panel) monitored by the cleavage of substrate LEHD-AFC showed a very good agreement with the iCasp9 dimerization observed by the decrease of iCasp9-mCherry signals via time lapse confocal imaging (black curve in right panel). Left panel shows a schematic view of the detection principle of Ac-LEHD-AFC system. **b,** Fluorescence signals of iCasp9-mCherry and microtubule-mCherry in cells after adding 0.25 nM inducer AP20187. **c,** Fluorescence signals of iCasp9-mCherry in iCasp9 cells treated with and without 0.25 nM inducer AP20187. Error bars represent ± standard deviation. **d,** Time sequence images of iCasp9 cell after adding 0.25 nM inducer AP20187. Top, CFP channel; Middle, FRET channel; Bottom, CFP and FRET merge channel. FRET Ratio is defined as the ratio of CFP intensity versus FRET intensity. **e** and **f,** Microscopy images (left panel) and flow cytometry analysis of FRET Ratio (right panel) for iCasp9 cells without inducer AP20187 treatment (**e**), and treated with 0.25nM AP20187 (**f**). Related to Fig 1

**Supplementary Fig. 2 Threshold setting for initial iCasp9 level.** **a,** Threshold of initial iCasp9 level set to distinguish survival cells with high initial iCasp9 level from those with low initial iCasp9 level. **b,** Initial iCasp9-mCherry level in survival and dead cells after the first (initial population) and third round (post-selection) of inducer treatment. The boxes of the box plot represent the interquartile range between the first and third quartiles, whereas the whiskers represent the 95% and 5% values, and the squares represent the average. Related to Figure 3 and Figure 6


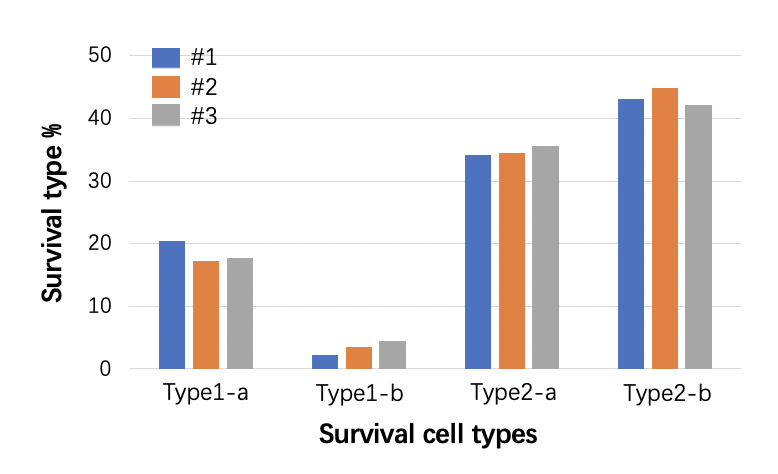


**Supplementary Fig. 3** **Percentage of different surviving cell types.** Different surviving cell types analyzed from three independent experiments. Related to Figure 3


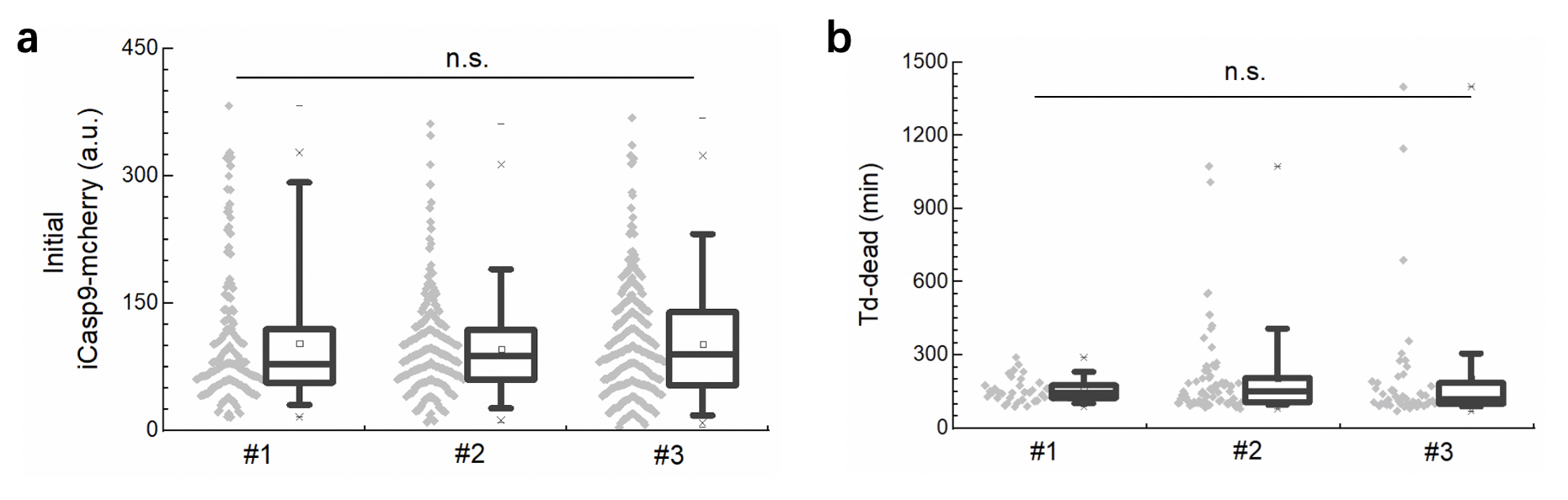


**Supplementary Fig. 4** **Initial iCasp9-mCherry level and Td-dead measured from three independent experiments.** **a,** Initial iCasp9-mCherry level in single cells treated with 0.25nM inducer from three independent experiments. Each dot represents one single cell. **b,** Td-dead in single cells treated with 0.25nM inducer from three independent experiments. Each dot represents one single cell. Related to Figure 4

**Supplementary Fig. 5 Characterization of cell death percentage. a,** Death percentage of iCasp9 cells after the addition of 0 nM, 0.025 nM and 0.25 nM inducer AP20187 for 12 h, 24 h and 48 h, respectively. **b,** Death percentage analyzed based on cell morphology and the FRET Ratio. Data are shown as mean ± standard deviation. Related to Figure 5

**Supplementary Fig. 6 Averaged dynamics of iCasp9-mCherry in survival cells with iCasp9 dimerization and death percentage under different concentrations. a,** Averaged dynamics of iCasp9-mCherry in survival cells with iCasp9 dimerization in condition of 0.025 nM, 0.25 nM and 2.5 nM inducer. Error bars represent ± standard deviation. **b,** Death percentage is plotted against Td-iCasp9. Data are shown as mean ± standard deviation. Related to Figure 5
